# The Rocky Road to Rejection Resilience: A Personal Publishing Journey

**DOI:** 10.5334/pme.1727

**Published:** 2025-09-11

**Authors:** Lynette Jean van der Merwe, Gina Joubert

**Affiliations:** 1 Faculty of Health Sciences, University of the Free State, Bloemfontein, South Africa

## Abstract

**Introduction:**

The journey through submission, rejection, and eventual publication of scholarly work is challenging to academic researchers’ resilience. Dealing with rejection without succumbing to burnout or impostor syndrome requires a growth mindset. This paper analyses one author’s manuscript rejections over five years and makes recommendations for academic researchers regarding manuscript rejections.

**Methods:**

This retrospective longitudinal mixed-methods study included one author’s rejected submissions from 2019 to 2023. Quantitative data on manuscript rejection characteristics: number of rejections, subsequent publication, submission (field and research type), journal location and impact factor, and nature of rejection (desk rejection, rejection after review or revision) were analysed descriptively. Qualitative data (narrative text indicating reasons for desk rejection) were analysed thematically. Ethics approval was obtained.

**Results:**

Eighty submissions of 47 manuscripts were rejected, including 65% desk rejections. Most manuscripts were rejected once (60%) or twice (26%), and 77% were subsequently published. Most submissions were to journals in Africa (56%), on postgraduate student research (63%), in the field of medicine (71%). Themes related to reasons for desk rejection included not meeting journal requirements (scope, focus, criteria or priority), manuscript inadequacy (novelty, relevance, methodology, or contribution), and ethical issues (similarity indices, or ethics documentation).

**Discussion:**

This study on manuscript rejections received by one author over five years revealed that most rejected manuscripts were subsequently published. Desk rejection was most common. We support literature on normalizing and destigmatizing rejection and bolstering resilience to support academic researchers when dealing with technical, manuscript-related revisions and inevitable emotional responses to rejection to ensure healthy longevity in their scholarly careers.

## Introduction

The adage “publish or perish” still holds power over academic researchers [1] who often also have teaching and supervisory responsibilities. This paper offers a unique contribution from an individual author’s experience of the publication journey. Although rejection of submitted manuscripts is a common, non-fatal feature of scholarly publication pursuit, authors’ responses may include abandoning the further pursuit of the publication or even a stymied professional career [2,3]. The laborious and lengthy process of manuscript submission and eventual publication of scholarly work is but one of the aspects of an academic researcher’s career. Overcoming rejection is integral to the academic publishing journey, as it is inevitably accompanied by negative emotions, ranging from shame to defeat [4].

Authors use various coping strategies to deal with the adverse reactions of manuscript rejection, requiring individual empowerment and institutional awareness about emotions associated with rejection experiences [3,5]. Repeated rejection, burnout, and impostor syndrome, although familiar to the academic researcher’s life, are often endured alone and shamefully, leading to a sense of isolation and stigmatization [6].

Rejection resilience has been described in academics who overcome negative emotions like shame, disillusionment, isolation, discouragement, or defeat due to multiple rejections of submitted manuscripts [7]. Academic researchers need rejection resilience to withstand the discouragement associated with multiple unsuccessful manuscript submissions [7]. Resilience is defined as the individual ability to bounce back after experiencing stress or negative emotions following rejection [8]. By drawing from psychological research, academic medicine has acknowledged the value of resilience as a framework to overcome the ubiquitous experience of professional rejection [6], recognizing resilience as a dynamic construct dependent on both individual and organisational resources and interventions [9].

Desk rejections by the journal editorial team with little or no feedback to authors are the first hurdle in publishing a scholarly manuscript and may disadvantage authors if the process is not fair, ethical and valid [10]. Multiple submissions before publication are standard, possibly contributing to delayed knowledge dissemination or author discouragement [1,3,7,10]. Novice authors need proactive and intentional mentoring in responding to editorial decisions to destigmatize rejection and foster a growth mindset toward resubmission and publication success [10,11].

As academic researchers involved in both undergraduate and postgraduate research teaching and supervision, based on their own experience, and literature regarding the publication process and reasons for manuscript rejection, including ineffective study design and execution, inadequate manuscript preparation, inappropriate journal selection or non-adherence to instructions, and concerns regarding scientific conduct [5,12,13], the study addressed the following overarching research question: How can patterns of manuscript rejection and the content of editorial feedback, as experienced by one academic author over five years, inform strategies to support researchers in navigating rejection and sustaining scholarly resilience? The study aimed to examine five years of manuscript rejection experiences by a single academic author through a mixed-methods approach including 1) a quantitative analysis of submission and rejection patterns and subsequent publication outcomes, 2) a qualitative thematic analysis of editorial feedback, particularly desk rejections, and 3) the development of recommendations to support academic researchers (especially novice researchers) in navigating rejection and sustaining scholarly resilience.

Through systematic analysis of rejection patterns and editorial feedback this study may contribute to a more transparent and humane culture of scholarship. The findings have practical utility for novice researchers seeking to navigate the publication process, for supervisors aiming to provide effective guidance and for journal editors interested in improving feedback practices. More broadly this work helps to normalise rejection as an integral part, rather than exceptional part, of academic life fostering resilience and sustainability in scholarly careers.

## Methods

This retrospective longitudinal mixed-methods case study focused on all rejections of manuscript submissions by one author for the period 2019 to 2023 interpreted through both descriptive statistics and thematic analysis of editorial feedback. This author, an experienced researcher, was the first or co-author of all the submissions included in the study. Other authors (whether first or co-authors) included both experienced and novice researchers (including undergraduate and postgraduate students). All manuscripts submitted for publication and reported on in this paper were deemed appropriate and important to publish, even though the findings reported on research by inexperienced authors. If a manuscript was rejected by more than one journal, each was considered a separate submission.

Correspondence from journals were retrieved from emails received by the authors or medical editors of the Faculty of Health Sciences or from the journal platforms in an Excel worksheet. Quantitative and qualitative data were collected concurrently and complementary triangulation of findings from descriptive statistical analysis of quantitative data and explanatory insights from editorial text (qualitative data) were compared and integrated. The second author noted quantitative data, including characteristics of the submission (field and research type), the journal (based in South Africa, other African countries, or elsewhere and journal impact factor [JIF]), and the rejection (desk rejection or sent for review, reason for rejection, any suggestion made). It was also noted whether the manuscript had subsequently been published, or publication was no longer pursued.

The second author summarized quantitative results by frequencies and percentages (categorical variables) and medians and interquartile ranges (numerical variables due to skew distribution) using SAS Version 9.4. Acceptance per submission and acceptance per manuscript rates proposed by Slattengren et al. [13] were calculated. Journal impact factors by subgroups were analysed using Kruskal-Wallis tests. The association between journal base and desk rejections was assessed using the chi-squared test. The level of statistical significance was 0.05.

The narrative text from journal editorial teams stating reasons for desk rejections (qualitative data) were analysed according to Braun and Clarke’s six steps in thematic analysis of qualitative data [14]. The first author became familiar with the data, and generated initial codes, categories for codes, sub-themes and themes. The themes were reviewed by both authors, and after defining and naming the themes, these were written up.

Reflexivity entailed recognizing the positionality of the researchers in this study as authors or co-authors of all the manuscript submissions, therefore analysing their own rejected manuscripts. The first author was involved in five of the 80, while the second author was involved in all 80 manuscript rejections. Through iterative discussions and debriefing during the submission process of this paper, the authors used reflexive research collaboration [15] to examine challenges to their resilience through the dynamics of repeated submissions and rejections of a paper on the topic of rejection resilience.

This project was approved by the Health Sciences Research Ethics Committee of the University of the Free State (UFS-HSD2022/0001/2202) Editorial correspondence used in this study was restricted to decision letters and desk rejection communications received by the authors. All identifying information about journals, editors and reviewers was removed. Quotations were anonymized to prevent reidentification, and data were securely stored on institutional servers with restricted access.

## Results

From 2019 to 2023, eighty submissions of 47 manuscripts were rejected. As indicated in Table 1, most manuscripts were rejected once (28/47 = 60%) or twice (12/47, 26%). Seventy-seven percent (36/47) of the rejected manuscripts were subsequently published, three of these by the journal that had initially rejected them (one a desk rejection, two rejections after review). One of the three was published as a revised and shortened letter to the editor (as suggested by the editor upon rejection of the manuscript), one as a revised full-length manuscript after an appeal to the editors and one (the initial desk rejection with editor’s suggestion to refocus the manuscript) after the unchanged manuscript was erroneously uploaded onto the journal submission platform again and subsequently reviewed and revised. The publication of nine manuscripts has been abandoned; six of these after the first submission was unsuccessful due to reviewer feedback, which identified unresolvable gaps or flaws in the methodology. Other reasons for abandoning publication were that no suitable journal could be found, and that the data were no longer current due to the time lapsed. Of the seven manuscripts that were rejected three or more times, four have been published, and the publication of one is still being pursued. During this period, ninety-three submissions were successful, giving an acceptance per submission rate of 53.8%. The acceptance per manuscript rate was 89.4%.

**Table 1:** Number of rejections per manuscript during 2019–2023 and subsequent publication status (n = 47).


	NUMBER OF REJECTIONS PER MANUSCRIPT	PUBLICATION ABANDONED	PUBLICATION STILL BEING PURSUED	PUBLISHED AFTER REJECTION

	*n(%)*	*n(%)*	*n(%)*	*n(%)*

1	28 (60)	6* (21)	1 (4)	21 (75)

2	12 (26)	1 (8)	0	11 (92)

3	2 (4)	0	0	2 (100)

4	3 (6)	2 (67)	0	1 (33)

5	2 (4)	0	1 (50)	1 (50)


* Three of these related to undergraduate student research projects and three to Master of medicine research; reviewer comments indicated that there were major methodological issues.

Table 2 provides the context of the characteristics of the submissions. Submissions were mainly to South African-based journals (43/80, 54%) in the field of medicine (57/80, 71%). Most submissions included junior authors reporting on student research including postgraduate Master of Medicine (medical specialization) (31/80, 39%), PhD or Master’s degree (19/80, 24%), and undergraduate medical student projects (16/80, 20%). Rejections came from journals with a median impact factor of 1.5 (IQR 1.2 to 2.5). All journals appear on accredited lists, including Web of Science, Scopus, Directory of Open Access Journals, and the South African National Government Department of Higher Education and Training accredited journals list [16]. Manuscripts published after rejection appeared in journals with a median impact factor of 1.2 (IQR 0.8 to 1.9).

**Table 2:** Submission characteristics (n = 80).


	n (%)

**Type of Research project**	

Master of Medicine (medical specialization)	31 (39)

Other postgraduate (Masters, PhD)	19 (24)

Undergraduate medical students research module	16 (20)

Staff (own research not for a qualification)	14 (17)

**Journal base**	

South Africa	43 (54)

Elsewhere in Africa	2 (2)

Outside Africa	35 (44)

**Field**	

Medical	57 (71)

Allied Health/Health and Rehabilitation Sciences	9 (11)

Health Professions Education	8 (10)

Other	6 (8)


Table 3 presents rejection characteristics. Most were desk rejections (52/80, 65%). Minimal rejections came after the manuscript was revised (4/80, 5%). Of the twenty-two submissions rejected after review (28%), the editor/associate editor gave guidance regarding the main concerns in only four cases (18%).

**Table 3:** Rejection characteristics.


REJECTION TYPE	ALL REJECTIONS (n = 80)	REJECTIONS FROM SA AND OTHER AFRICAN JOURNALS (n = 45)	REJECTIONS FROM JOURNALS OUTSIDE AFRICA (n = 35)

	n (%)	n (%)	n (%)

Desk rejection (not sent for review)	52 (65)	26 (58)	26 (74)

Rejected, unclear whether sent for review	2 (3)	1 (2)	1 (3)

Rejected after review	22 (28)	14 (31)	8 (23)

Editor gave guidance regarding main concerns	4 (5)	1 (2)	3 (9)

Rejected after revisions	4 (5)	4 (9)	0


Field and project type were not significantly associated with desk rejections (p = 0.61 and p = 0.55 respectively). The highest percentage of desk rejections was for staff projects (11/14 = 79%) and the lowest for undergraduate medical student research projects (9/16, 56%). With respect to field the percentage of desk rejections ranged from 56% (5 out of 9 rejections) for Allied Health/Health and Rehabilitation Sciences to 88% for Health Professions Education (7 out of 8 rejections). Desk rejections were more common in journals outside Africa than in African and South African-based journals, but the differences were not statistically significant (p = 0.12). There was a significant downward trend (p = 0.04) in journal impact factors (JIF) from desk rejections (median JIF 2.2), rejection after review (median JIF 1.4), to rejection after revision (median JIF 1.2).

Table 4 gives an overview of the thematic analysis of desk rejections. Of the fifty-two desk rejections, correspondence for one could not be traced and was indicated “unknown.” The remaining text in fifty-one desk rejections was coded and categorized as indicated. Categories referred to the comprehensiveness of feedback (brief or expanded) and whether it included any guidance for the authors or reasons for the desk rejection. Sub-themes were identified based on the feedback that was either generic (automated/stock text) or specific (referring to the submission content). Finally, three themes were identified, namely, 1) The journal requirements were not met, 2) The manuscript was considered inadequate, and 3) There were ethical issues identified.

**Table 4:** Thematic analysis of reasons for desk rejections of submitted manuscripts.


THEMES	DESCRIPTION OF THEME	SUB-THEMES	CATEGORIES	CODES

1. Journal requirements not met	Feedback to authors was that the submission was rejected based on the journal’s requirements regarding scope, focus, criteria, or priority	Generic feedback Specific feedback	Brief Expanded Guidance/reason	Scope, focus, criteria, priority

2. Manuscript considered inadequate		Generic feedback Specific feedback	Expanded Guidance/reason	Novelty, relevance methodology, contribution, references

3. Ethical issues identified		Specific feedback	Reason	Similarity index, ethics documentation


The first theme, **the journal requirements were not met**, was the most common reason for thirty-seven (73%) of the desk rejections. Most reasons were generic and brief (n = 21, 57%), for example, ‘not suitable for the journal,’ and ‘not within the journal’s scope and focus.’ Of note, thirteen of the desk rejections due to not fulfilling the journal scope were from one general medical journal based in South Africa. Expanded generic responses (10, 27%) revealed that although more detailed feedback was given, it remained non-specific, not guiding the authors regarding where their submission fell short. Examples included that the submission’s subject matter and general content were not suitable

‘All new manuscripts are given a preliminary review by the editor(s) to assess whether the subject matter and general content are appropriate for this journal. Unfortunately, the editor(s) felt that your submission should be rejected without an external review, and we are therefore returning your manuscript,’

or that the submission did not meet a variety of criteria, although none were highlighted

‘does not meet our criteria for publication. Major issues that commonly reduce a manuscript’s priority for publication in the xxx [journal name redacted to ensure confidentiality] include lack of novelty, limited significance and/or impact, methodological and/or statistical concerns, and over-interpretation of the data,’

In some of these expanded responses, there seemed to be missing text, possibly indicating auto-generated responses with no editorial input, as per the following example, with no subsequent text.

‘Unfortunately, it seems very unlikely that any revisions could be made that would render the manuscript acceptable for external review in this journal because:’ [no further text available]

Specific reasons or guidance were stated in seven (19%) responses under the theme **the journal requirements were not met**. For example, one journal indicated that the submission fell short due to its localized setting, limiting its relevance internationally

‘it does not meet the criteria for xxx [journal name redacted to ensure confidentiality]. Studies reporting descriptive results from a single institution or region will only be considered if data and the conclusions provide distinct insights that are of relevance to an international audience. Your study focuses on the exploration and evaluation of a local teaching situation. Hence, criteria for xxx [journal name redacted to ensure confidentiality]. have not been met’.

Another example offered the author(s) clear feedback towards improving subsequent submissions of the manuscript

‘does not relate sufficiently to the aims and scope of xxx [journal name redacted to ensure confidentiality]. While the findings of your survey are interesting, they are largely descriptive and for our journal we would expect a more rigorous discussion of the implications of the findings in relation to criminological or forensic psychology’

Furthermore, author(s) were encouraged that while their work was of good quality, it was not necessarily appropriate for the journal in its presented format, as seen in the below responses.

‘at the current time not appropriate for the journal. We felt you did an excellent job doing what you set out to do in describing the demographic and clinical profile of your adolescent patients. However, in doing so, you failed to offer insights into the broader social and environmental context that give rise to and sustain such behaviours. Whilst we recognise the importance of descriptively profiling patients, we believe you will make a more significant contribution, both to the clinical care youth and to the field as a whole if you help readers better understand the role that social, environmental, and cultural influences have on minority and otherwise marginalised youth’s mental health and wellbeing.’

The second theme, namely, **the manuscript was considered inadequate**, occurred in fifteen (29%) desk rejections. In most cases, the response was specific with reasons or guidance, providing the author(s) with constructive feedback. Issues with methodology were commonly stated as reasons for desk rejection (n = 10, 20%). Some were cryptic, for example,

‘While the topic is of interest, the retrospective nature of the analysis and the small patient number preclude any meaningful conclusion.’

Other responses were more extensive, for example,

‘My decision is based on my belief that your study would be strengthened considerably by the inclusion of more data that spans multiple sites in order to provide more convincing evidence of the robustness and generalisability of your findings,’

or directive, for example,

‘methods described in the manuscript are insufficient to reliably assess the association between sickness absenteeism. The methods should consider issues with statistical power (use fewer meaningful categories for the exposure variable) and address confounding. It is also important to clearly define the exposure variable. Using absolute changes in BMI as the exposure variable might make the analysis easier for you and your team. Moreover, please highlight how you dealt with missing data in the analysis.’

Under the theme, namely, **the manuscript was considered inadequate**, seven (7/15 [47%]) desk rejections stated reasons related to the relevance and novelty of the findings or contribution. For example, the rejection of small, single-site studies with limited scholarly rigour

‘In reaching an editorial decision, the Editors consider the scientific quality of the manuscript, the novelty of the observations, and the relevance for our readership. Unfortunately, a survey of medical students at just one school is not generalizable and does not make a significant enough contribution to the research literature.’

offered the authors explicit insight into editorial decisions and priorities. Similarly, an apparent lack of novelty led to rejection, as evidenced by this response:

‘Editorial team considers that the material or subject matter presented in this manuscript has been previously published in this, or another journal in the setting of a larger number of patients or with very similar results. In our aim to prioritize manuscripts of novel research, we have collectively determined not to submit this manuscript for further review.’

Additionally, editors mentioned issues related to literature, e.g.. ‘outdated literature search’ and referencing

‘the reference list to be updated with most recent sources before the article will be considered fitting for review.’

However, there were no rejections due to language or journal submission guidelines (administration and quality) not being met.

The third theme, **ethical issues identified**, was least frequently noted (n = 4, 8%). The specific reasons stated included, for example, regarding similarity indices:

‘The similarity index is 71%. The authors are invited to rework the article to an index of 35% or less and acknowledge the origin of the works’

and issues with ethics documentation:

‘The ethics certificate is dated 2019 – but the data were collected the previous year.’

## Discussion

This study investigated eighty desk rejections of 47 manuscripts experienced by one author over five years. Quantitative data analysis revealed that despite multiple rejections most articles were subsequently published. Thematic analysis of narrative text highlighted reasons for desk rejection although feedback to authors was rarely informative.

Rejection rates vary from 60–90% [1,22], and up to 62% of published articles are reportedly initially rejected [19]. Allen et al. [7] have reported that 80% of Paediatric academic staff have had to submit a manuscript to three or more journals before acceptance. This study’s acceptance per submission rate (53.8%) was similar to the 53% reported among academic clinicians in Paediatrics but lower than the 69% reported among Family Medicine clinicians [13]. The acceptance per manuscript rate in this study (89.4%) was, however, higher than the 76% and 83% reported by Paediatrics and Family Medicine clinicians, respectively [13], possibly due to perseverance to submit a manuscript that is considered worthwhile until it is accepted. Although exposure to the publication process is valuable research training for students, at out institution publication is only considered if the manuscript merits publication. Publication of undergraduate medical student research projects is only pursued in cases where the supervisor and/or module leader consider the material suitable for publication in journals of standing. The students are the first authors listed on their full project report if the report has only been adapted editorially for journal submission. If the supervisor had to change the report substantially for submission (usually the Introduction and Discussion), the supervisor could become the first author. Students are fully informed about this approach, review and approve the final version for publication, and are kept informed about the submission process. Although publication of postgraduate projects is not a requirement, the rigorous screening by Faculty evaluation committees, the institutional ethics review committee, and feedback from external examiners ensure that many are considered worthy of pursuing publication. Postgraduate students are the first authors of such submissions. Finding a good journal fit for such manuscripts depends on the topic and local or broader interest.

Reasons for desk rejections in this study are similar to those listed in an editorial by Dwivedi et al. [11], including scope and lack of alignment, research contribution and novelty, and theoretical and methodological issues. This study did, however, not find administration and quality as reasons for rejection, reflecting meticulous preparation and adherence to author guidelines. The most common theme identified for desk rejections was that the submission did not meet the journal requirements despite authors considering the author guidelines (including scope) before submission. The decision to submit a manuscript to a local (South African), African or international journal was based on the topic, the impact of the findings, and the availability of appropriate journals. Authors need to study journal scopes closely and journal editors must ensure that journal scopes are explicit and that rejection decisions align with the stated journal scope. In most cases, the reasons stated were generic and brief, possibly auto-generated, and provided no feedback to the author(s), disadvantaging novice researchers who may rely on editorial guidance. Where generic reasons were expanded, feedback was somewhat more directive, although nebulous. Some responses also seemed auto-generated. For example, the text mentioned that reasons for the desk rejection would be given, but none were stated. Where specific and expanded feedback was given, although there may have been reassurance about acceptable quality, resubmission to the same journal would most likely be futile [17,18].

The theme related to the adequacy of the submitted manuscript highlighted issues related to novelty, methodology, relevance, and the contribution of findings. Where reasons for rejection referred to methodology, these could guide the authors in preparing revised manuscripts for subsequent submissions. In some cases, limited generalizability of the reported study’s findings would preclude publication even upon subsequent review. Still, in other instances, the specific feedback related to the analysis or presentation of findings provided the author(s) with constructive suggestions for revising the manuscript for ensuing submission attempts. Authors may avoid desk rejection by addressing such issues before submission as a part of quality assurance. Although the research process is considered incomplete if findings are not published, one must also consider that some projects are primarily performed to expose novice researchers to research. Also, if reviewers highlight severe methodological issues, investing even more time in pursuing publication is not worthwhile.

Repeated submissions of rejected manuscripts are time-consuming [2]. Feedback from the editorial team can support authors in preparing subsequent submissions of articles rejected after review to avoid further delays in publication. In this study, where a third of submissions were rejected after review, editors provided feedback on specific reasons for rejection in only a few instances.

Issues related to ethics were not commonly stated as reasons for desk rejection in this study, reflecting sound research practices. The two rejections regarding the similarity index were related to publications from doctoral degrees written in article format, illustrating a tension between open-access dissertation mandates and journal originality checks. Journal guidance in this regard was not apparent at submission. In both cases the authors communicated with the editors after the rejection and both editors required that the manuscripts be rewritten substantially so that there was less similarity to the source doctoral theses. Clearer guidelines and education for researchers and authors about self-plagiarism may be valuable. The authors would have quickly clarified the issues relating to ethics documentation if they had been given the opportunity to respond.

Novice authors need support to grow rejection resilience, besides guidance on the technical aspects of manuscript submission or revision after rejection [3,10,11]. Paying close attention to feedback received from the editor to ensure that the following submission (whether to the same or another journal) meets research and technical requirements is fundamental [2,19]. For this to be possible, journal feedback should preferably be specific and non-generic. The nature of feedback reported in this study demonstrated limited value in guiding authors in revising their manuscripts.

Clear guidance from journals would be of value for all researchers. Most of the rejected manuscripts in our study were related to student research (postgraduate or undergraduate), implying that the publication process was a new experience for the researchers. Although supervisors were available to accompany these novice researchers through this aspect of the research process, explicit statements from journals would be especially beneficial to these researchers. Dealing with the emotional response to rejection may require additional support and skills [3,4].

The experience of preparing, submitting, and receiving multiple rejections of this manuscript for publication on the topic of manuscript rejections required resilience from both authors. The study reports on publication experiences of mostly novice researchers (undergraduate and postgraduate research projects). Therefore, in the light of our experience, the study findings and published literature on the publication journey, we support considering the following when dealing with manuscript rejection:

- Normalization. Authors should have realistic expectations when submitting their manuscripts based on high initial rejection rates in a competitive academic publishing environment [6] and subsequent successful publication in other journals [13]. As was experienced in this publication journey, authors should include multiple cycles of submission, rejection, revision, and resubmission in their planning and time management [5,10]. Practical guidance, e.g., the flow diagram proposed by Woolley and Barron [20], is an excellent example of a simple approach to responding to rejections.
- Destigmatization. Destigmatizing rejection has been proposed by sharing a failure *Curriculum Vitae* [CV] or authors publicly revealing their cycles of submission, rejection, and acceptance to peers [3,21], as we have done in this study. Alternative metrics (beyond citations or H-indices) focusing on the individual researcher’s self-assessment of acceptance rates per submission and eventual acceptance rates may be worth exploring [13].
- Bolstering resilience. The pervasive critique and competition in the academic medical environment, exemplified in manuscript rejections, emphasizes the need for resilience [2,3,6,22]. Individual researchers could focus on using adaptive coping strategies, including dealing with emotions positively and cognitive reframing [2,6,20,22]. For example, as authors of this paper, we tried to maintain a growth mindset reframing multiple submissions, rejections and re-submissions as learning experiences contributing to resilience. Mentoring systems that offer moral support and encouragement and promote positive thinking and adaptive mindsets are vital for sustainable academic careers characterized by rejection resilience [6].

## Limitations

The generalizability of this study’s findings is limited as it investigated the experiences of manuscript rejections by one author in a health sciences faculty at a South African institution. This study could not investigate factors associated with different types of rejections due to its sample size and profile of manuscripts. Nevertheless, the findings concur with literature describing manuscript rejections (usually referring to specific journals or study fields) and offer a unique contribution from an individual author’s experience.

## Conclusions

This study on manuscript rejections received by one author over five years revealed that most rejected manuscripts were subsequently published. Desk rejection was the most common type of rejection. The themes related to desk rejection included the manuscript not meeting journal requirements (scope, focus, criteria, or priority), manuscript inadequacy (novelty, relevance, methodology, or contribution), and ethical issues (similarity indices or ethics documentation). By knowing the reasons for rejection experienced by other authors and considering suggestions, including normalizing and destigmatizing rejection and bolstering resilience, academic researchers could be supported when dealing with technical, manuscript-related revisions and inevitable emotional responses to rejection of manuscript submissions to ensure healthy longevity in their scholarly careers. The findings have practical utility for novice researchers seeking to navigate the publication process, for supervisors aiming to provide effective guidance and for journal editors interested in improving feedback practices. More broadly this work helps to normalise rejection as an integral part rather than exceptional part of academic life fostering resilience and sustainability in scholarly careers. Additionally, the findings suggest that journals could adopt more developmental editorial practices to offer clarity and encourage revision rather than rejection as default. Further research comparing rejection resilience among novice and experienced academic researchers in the face of contextual disruptions, including artificial intelligence, could be valuable.

## Data Accessibility Statement

The datasets used and/or analysed during the current study are available from the corresponding author on reasonable request.

## Author Information

Both authors (LJvdM and GJ) are academic researchers involved in supervising undergraduate and postgraduate student research. This research was conceptualized from personal and professional experience and aims to support researchers in the arduous and competitive process of publication that is an integral part of a scholarly career.
